# Retrospective Analysis of IOL Power Calculation by Ray Tracing in Eyes with Previous Radial Keratotomy

**DOI:** 10.3390/jcm15020866

**Published:** 2026-01-21

**Authors:** Giacomo Savini, Kenneth J. Hoffer, Arianna Grendele, Catarina P. Coutinho, Andrea Russo, Domenico Schiano-Lomoriello

**Affiliations:** 1 IRCCS Bietti Foundation, 00184 Rome, Italy; domenico.schiano@fondazionebietti.com; 2 St. Mary’s Eye Center, Santa Monica, CA 90404, USA; khoffermd@startmail.com; 3 Stein Eye Institute, University of California, Los Angeles, CA 90095, USA; 4 Studio Oculistico d’Azeglio, 40123 Bologna, Italy; grendele.arianna@gmail.com (A.G.); catarina.praefke@gmail.com (C.P.C.); 5 Dipartimento di Farmacia e Biotecnologie, Alma Mater Studiorum Università di Bologna, 40126 Bologna, Italy; 6 Centro Oculistico Bresciano, 25122 Brescia, Italy; dott.andrea.russo@gmail.com; 7 Department of Ophthalmology, Unicamillus-Saint Camillus International University of Health Sciences, 00131 Rome, Italy

**Keywords:** IOL power calculation, ray tracing, radial keratotomy

## Abstract

**Background/Objectives**: To evaluate the predictive accuracy of intraocular lens (IOL) power calculation by ray tracing in eyes with previous radial keratotomy (RK). **Methods**: A consecutive series of eyes with previous RK was retrospectively analyzed. Preoperatively, all eyes underwent optical biometry to measure the axial length (AL) and anterior segment imaging by the MS-39 (CSO), which combines Placido disk corneal topography and anterior segment optical coherence tomography. The built-in ray tracing software was used to calculate the IOL power. For comparative purposes, the results of the Barrett True-K, EVO, Haigis total keratometry, and PEARL-DGS formulas were also investigated. The refractive outcomes were evaluated with Eyetemis. **Results**: Twenty-four eyes (24 patients) were investigated. The mean AL and keratometry were, respectively, 27.34 ± 2.88 mm and 35.53 ± 3.66 diopters (D). The mean prediction error (PE) was −0.03 ± 0.65 D (range: from −1.30 to +1.64 D). The mean and median absolute errors were 0.52 and 0.48 D, respectively. The percentages of eyes with a PE within ±0.25 D, ±0.50 D, and ±1.00 D were 29.17%, 62.50%, and 87.50%, respectively. A comparison with the other formulas was possible in 20 eyes and did not reveal any statistically significant differences; the percentage of eyes with a PE within ±0.50 D ranged from 50 to 65%. **Conclusions**: Ray tracing is a relatively accurate solution for calculating the IOL power in eyes with previous RK. Paraxial formulas provide similar outcomes and should be considered in these patients. The refractive outcomes of IOL power calculation in post-RK eyes are still below modern benchmarks for virgin eyes.

## 1. Introduction

Intraocular lens (IOL) power calculation in eyes with previous radial keratotomy (RK) is still a major challenge for cataract surgeons. In addition to the well-known sources of errors typical of eyes that have undergone excimer laser refractive surgery (i.e., the instrument error, the index of refraction error, and the formula error) [1], other important factors reduce the accuracy of IOL power calculation after RK. First, the optical zone is often small, irregular, and decentered; this makes it difficult to accurately measure the anterior corneal radius, due to the fact that keratometry (K) mires are projected onto the area representing the inflection point between the incised cornea and the flattened central region. Second, the irregularity of the optical zone can generate higher-order aberrations (HOAs) of the cornea, the effects of which can hardly be taken into account by paraxial IOL formulas. Third, corneal flattening of both corneal surfaces can be much higher [2], so some formulas cannot be used (the Barrett True-K, EVO, and PEARL-DGS, for example, do not allow users to enter keratometry (K) values lower than 30 diopters (D)). Fourth, RK encompasses cases with different numbers of incisions leading to different degrees of flattening, which makes it hard to develop predictive models. Finally, eyes with prior RK are relatively rare compared with those that have undergone myopic LASIK or PRK and are prone to early hyperopic shift and diurnal variations [3].

For these reasons, the results of IOL power calculation after RK are poor compared with both calculations in virgin eyes and in eyes with previous excimer laser refractive surgery. Most studies have reported that, even with the best formulas, only about 50% of eyes show a prediction error (PE) within ±0.50 D [4,5,6,7,8,9], a percentage remarkably lower than those observed in unoperated as well as in post-LASIK eyes. Modern formulas, in fact, can lead to good refractive outcomes after laser vision correction, with more than 70% of eyes achieving a PE within ±0.50 D when the Barrett True-K, EVO, Hoffer QST, or PEARL-DGS are used [10,11]. The only exception is a paper by Turnbull et al., who found that 76.6% of eyes could achieve a PE within ±0.50 D with the Barrett True-K if the preoperative refraction was known [12].

Exact ray tracing offers a potentially useful solution for calculating the IOL power in post-RK eyes because it considers both corneal surfaces (and, therefore, is not affected by the keratometric index error), takes HOAs into account, can be adjusted according to the pupil size, and does not need historical data. We previously found that the ray-tracing software available on the anterior segment optical coherence tomographer (AS-OCT) MS-39 (CSO) leads to accurate refractive outcomes after myopic LASIK [13]. In this study, we aimed to assess its performance in eyes with previous RK.

## 2. Materials and Methods

This was a retrospective analysis of consecutive eyes that underwent phacoemulsification and IOL implantation after previous RK at two institutions, Studio Oculistico d’Azeglio (Bologna, Italy) and Centro Oculistico Bresciano (Brescia, Italy), between 2019 and 2024. De-identified data were transferred to and analyzed at IRCCS Bietti Foundation (Rome, Italy). The study was conducted in accordance with the Declaration of Helsinki and approved by the Comitato Etico Centrale IRCCS Lazio (protocol code CEC/157/15, date of approval—31 March 2015). Informed consent was obtained from all subjects involved in the study. Exclusion criteria were any intraoperative or postoperative complications, postoperative corrected distance visual acuity equal to or lower than 20/40, and implantation of a pinhole IOL (as this may mask postoperative refractive errors due to the elongated depth of field). Eyes were not excluded based on the number of radial incisions or the time since RK. Subjective refraction was performed at 4 to 6 weeks postoperatively, as previously reported by other authors in post-RK eyes [12], at a distance of 4 m.

### 2.1. Preoperative Measurements and Surgery

The same procedure as previously described in our study on post-LASIK eyes was followed [13]. Briefly, patients underwent anterior segment imaging with the MS-39 (software version 4.1.4), which measures the anterior corneal curvature by means of AS-OCT and Placido disk topography and the posterior corneal curvature by means of AS-OCT. The same instrument was also used to assess the pupil diameter under photopic, mesopic, and scotopic conditions. The high repeatability of its measurements has been previously demonstrated [14]. Only scans with good quality, as assessed by the instrument, were used. Optical biometry was performed with the IOLMaster 700 (software version 1.90.38.02, Zeiss, Jena, Germany. Cataract surgery was performed by 3 experienced surgeons using a temporal clear corneal incision, phacoemulsification, and IOL implantation in the bag. In no case was a scleral approach deemed necessary in our series to reduce the risk of RK incisions opening. No intraoperative or postoperative complications occurred.

### 2.2. Intraocular Lens Power Calculation

The IOL power was calculated using the built-in ray tracing software available on the MS-39, which requires a few manual inputs: the AL, the target refraction, the pupil diameter, and the IOL A-constant. As regards the pupil diameter, since 3 diameters are available (2.0, 2.5, and 3.0 mm), the value closest to the one measured by the MS-39 was selected. The A-constant was retrieved from the User Group for Laser Interferometry Biometry (ULIB, www.ocusoft.de/ulib/c1.htm) or the IOLCon websites (https://iolcon.org, both accessed on 14 November 2025). The A-constant is needed to refine the prediction of the IOL position through a proprietary method. The AL measured by the IOLMaster 700 was used. Based on our previous study [13], the AL value obtained by optical biometry was adjusted according to the polynomial equation described by Holladay for the Holladay 2 formula in the errata accompanying the letter by Wang et al. [15]. This adjustment, which was carried out using Holladay IOL Consultant Software and Surgical Outcomes Assessment (version 2022.0910, Bellaire, TX, USA), aims to avoid the AL overestimation (and subsequent IOL power underestimation) typical of optical biometry in long eyes [15]. The ray tracing software is based on Snell’s law and specific refractive indices for each optical element. The method used to calculate the IOL power has been previously described in detail [13].

For comparative purposes, based on the IOLMaster 700 measurements, the IOL power was also calculated with the post-RK version of the Barrett True-K formula (www.apacrs.org), the EVO (www.evoiolcalculator.com), and PEARL-DGS (www.iolsolver.com, all accessed on 15 November 2025) [16]. Other new-generation formulas, like the Cooke K6, Hoffer QST, and Kane, could not be evaluated, as they do not offer a post-RK version. The Barrett True-K and EVO were used with both predicted and measured posterior corneal astigmatism (i.e., posterior keratometry (PK)). The constants available on the formula websites were adopted. In addition, total keratometry values from the IOLMaster 700 were entered into the standard Haigis formula [17], since this combination has been previously found to be accurate in post-myopic LASIK eyes [5].

The PE was calculated as the difference between the measured and the predicted postoperative refractive spherical equivalent for the power of the implanted IOL, so that a negative PE was correlated with a more myopic result than planned and a positive PE to a more hyperopic result. The mean PE, its standard deviation (SD), the median absolute error (MedAE), the mean absolute error (MAE), and the percentage of eyes whose PE was within ±0.25 diopters (D), ±0.50 D, ±0.75, and ±1.00 were calculated.

### 2.3. Statistics

MedCalc (software version 12.3.0.0, Ostend, Belgium) was used for statistical analysis. Correlation was used to evaluate the relationship between the PE and the preoperatively measured variables. While the primary aim of this study was to evaluate the accuracy of ray tracing for IOL power calculation after RK, a paired comparison was performed with the Barrett True-K, EVO, PEARL-DGS, and Haigis-TK for the subset of eyes whose IOL power could be calculated with these methods (i.e., those with an average K > 30 D). For this purpose, Eyetemis was used (www.eyetemis.com, accessed on 14 November 2025); this tool evaluates trueness, precision, and accuracy by means of robust *t*-tests [18]. The Cochran Q test was adopted to identify significant differences within a certain range of PE (e.g., within ±0.50 D).

Given a type I error of 0.05 and a power of 0.80, a mean difference in the PE of 0.50 D and a mean standard deviation of the difference of 0.70 D, PS Power and Sample Size (https://github.com/vubiostat/ps/, accessed on 20 January 2026) calculated a minimum sample size of 18 eyes. A *p*-value < 0.05 was considered statistically significant.

## 3. Results

Twenty-eight eyes of 27 patients with previous RK were identified. Four eyes of three patients with a pinhole IOL were excluded, thus leaving a dataset of twenty-four eyes of twenty-four patients (thirteen males (54%), mean age: 61.2 ± 8.2 years, mean number of RK incisions: 10.4 ± 3.1). Eight different IOL models were implanted, and the most commonly used were the Tecnis Ehyance (Johnson & Johnson, Irvine, CA, USA, n = 9), the toric AcrySof SN6ATx (Alcon, Ft. Worth, TX, USA n = 5), and the non-toric AcrySof SN60WF (n = 4). The mean biometric measurements are reported in Table 1. On average, standard keratometry underestimated total keratometry by 0.16 ± 0.20 D. In no case was the difference higher than 0.50 D, and in 16 eyes (64%), the difference was lower than 0.25 D.

Based on ray tracing, the mean PE was −0.03 ± 0.65 D (range: from −1.30 to +1.64 D). The MedAE was 0.48 D, and the MAE was 0.52 D. The percentage of eyes with a PE within ±0.25, ±0.50, ±0.75, and ±1.00 D was 29.17%, 62.50%, 87.50%, and 91.67%, respectively. All cases had a PE within ±2.00 D. When AL was <26 mm (n = 12), the percentage of eyes whose PE was within ±0.50 D was 75%, compared with 50% when it was >26 mm (n = 12). The PE was not correlated with AL (r = 0.05, *p* = 0.82), K (r = 0.35, *p* = 0.10), ACD (r = −0.38, *p* = 0.07), LT (r = 0.32, *p* = 0.14) or photopic pupil diameter (r = 0.17, *p* = 0.43).

The Barrett True-K, EVO, and PEARL-DGS formulas could not be used in four eyes whose average K was <30 D. In the remaining 20 eyes, the results (Table 2) were similar to those obtained with ray tracing, and Eyetemis did not detect any statistically significant differences for trueness, precision, accuracy, or for the percentage of eyes within each threshold. More specifically, according to the two-sample robust *t*-test, the adjusted *p*-value ranged between 0.58 and 1 for trueness, between 0.46 and 1 for precision, and between 0.12 and 1 for accuracy. According to the Cochran Q test, the adjusted *p*-values ranged between 0.27 (for the PE threshold of ±0.75 D) and 0.93 (for the threshold of ±1.00 D).

## 4. Discussion

Our study confirms that IOL power calculation after RK is still a challenging task, since the refractive outcomes are usually worse than those achieved not only in virgin eyes but also in eyes with previous myopic PRK or LASIK. In fact, in the event of previous myopic laser vision correction, most formulas lead to a PE within ±0.50 D in a percentage of eyes that ranges between 60 and 70% [11], but with the best formulas, the percentage can be higher than 70% [10,13,19]. In our dataset of post-RK eyes, the highest percentage was obtained with ray tracing, which reached 62.5% across the whole sample (n = 24) and 65% in the subset (n = 20) used for formula comparison (Barrett True-K, EVO, Haigis-TK, and PEARL-DGS). With the remaining formulas, only 50–60% of eyes resulted in a PE within ±0.50 D, with the exception of the Haigis-TK (65%). It should be highlighted that in post-myopic LASIK eyes, the same ray tracing technology enabled us to calculate a PE within ±0.50 D in a higher (77%) percentage of eyes [13]. Due to the retrospective nature of the study and the lack of postoperative AS-OCT measurements, we cannot assess whether the lower accuracy in post-RK eyes depends on an incorrect prediction of the postoperative IOL position, a postoperative change in corneal power, or other factors.

In addition to the highest percentage of eyes with a PE within ±0.50 D, ray tracing offered a mean PE close to zero, thus showing that the combination of optimized constants derived from ULIB or IOLCon datasets and AL optimization previously adopted by our group in post-LASIK eyes is also a good approach in post-RK eyes [13]. On the other hand, the refractive outcomes of the Barrett True-K, EVO, Haigis-TK, and PEARL-DGS did not show any statistically significant differences compared with ray tracing. All formulas had a PE < 0.30 D, and absolute PEs slightly higher or slightly lower than those obtained with ray tracing. As a consequence, none of these methods should be discarded a priori when calculating the IOL power in eyes with previous RK, unless the average K is lower than 30 D. Another interesting observation regards PK: as opposed to what has been observed in post-LASIK eyes [10,11], PK does not seem to significantly improve the accuracy of the Barrett True-K and EVO formulas. Although a certain explanation cannot be provided, since these formulas are unpublished and their structure is unknown, we can postulate that this result depends on the large variability of post-RK corneal curvature [2]. After myopic LASIK, we can assume that the posterior corneal curvature is unchanged and can therefore be used to estimate the pre-LASIK anterior corneal curvature, based on the anterior-to-posterior ratio of unoperated eyes. Once the pre-LASIK anterior corneal curvature is estimated, the double-K method can be applied [20]. This procedure is expected to improve the refractive outcomes of IOL power calculation. After RK, on the contrary, the posterior corneal curvature undergoes a remarkable and unpredictable flattening [2]. As a consequence, it cannot be used to estimate the pre-RK anterior corneal curvature, which is necessary to predict the IOL position by the double-K method.

With respect to previously published studies, our data suggest that ray tracing provides us with potentially accurate refractive outcomes. Voytsekhivskyy did not yield percentages of eyes with a PE within ±0.50 D higher than 53% [4]. None of the formulas investigated by other authors reached the value of 50% [6,7,9], except for the Barrett True-K and the ASCRS calculator average power, which obtained a PE within ±0.50 D in 56% of cases in a study by Shetty et al. [8]. However, the real exception is the paper by Turnbull et al., who found that more than 70% of eyes had a PE within ±0.50 D with the Barrett True-K [12]. This difference may depend on the lower AL of their sample, whose mean was 24.98 ± 0.87 mm as compared with an average of 27.33 ± 2.94 mm in our dataset [12]. Although we did not detect a statistically significant relationship between the PE and AL, our subgroup analysis revealed that eyes with a shorter AL (<26 mm) can reach a higher percentage (75%) than eyes with a longer AL (50%). We can therefore argue that IOL power calculation after RK is more accurate in shorter eyes and that studies with a lower mean AL are likely to display better outcomes.

This study has some limitations. First, the sample size was small, and for this reason, we aim to collect a larger sample. However, since—apart from a single case report—[21] there are no published papers about IOL power calculation by ray tracing in post-RK eyes, we feel that our results are important for both ophthalmologists and patients, who can at least have preliminary information on the accuracy of this method. Second, no postoperative scans were available for the majority of eyes, so we could not assess the influence of corneal power changes and errors in IOL position predictions. Third, different IOL models were included. Finally, slightly different results might have been obtained if ray tracing could have been performed through pupil diameters larger than 3.0 mm.

## 5. Conclusions

Our data show that ray tracing is a promising option to calculate the IOL power in eyes with previous RK, although the refractive accuracy is still lower than in eyes with previous laser vision correction, as well as in unoperated eyes. Modern theoretical formulas provide similar outcomes and should be considered for these patients.

## Figures and Tables

**Table 1:** Biometric measurements were obtained with the IOLMaster 700 in the 24 eyes analyzed.

	Mean ± SD	Range (min/max)
Keratometry flat (D)	34.59 ± 4.01	26.86/41.59
Keratometry steep (D)	36.57 ± 3.41	29.34/41.76
Keratometry average (D)	35.53 ± 3.66	28.08/41.68
Posterior keratometry flat (D)	−4.38 ± 0.66	−5.41/−3.1
Posterior keratometry steep (D)	−4.74 ± 0.50	−5.49/−3.82
Posterior keratometry average (D)	−4.54 ± 0.58	−5.45/−3.44
Total keratometry (D)	35.47 ± 3.67	28.25/41.83
Anterior chamber depth (mm) *	3.34 ± 0.33	2.67/3.93
Lens thickness	4.45 ± 0.35	4.01/5.2
Axial length	27.33 ± 2.94	23.42/33.13

* Measured from the corneal epithelium to the anterior surface of the lens.

**Table 2:** Comparison of IOL power calculation, according to ray tracing and other formulas, in 20 post-RK eyes.

	Mean PE ± SD (D)	MAE (D)	MedAE (D)	PE ≤ ±0.25 D	PE ≤ ±0.50 D	PE ≤ ±0.75 D	PE ≤ ±1.00 D
Ray tracing	0.01 ± 0.69	0.54	0.48	25%	65%	85%	90%
Barrett True-K P-PCA	−0.19 ± 0.74	0.56	0.44	35%	55%	80%	85%
Barrett True-K M-PCA	−0.05 ± 0.81	0.60	0.36	25%	60%	70%	80%
EVO P-PCA	−0.02 ± 0.74	0.53	0.35	40%	55%	70%	80%
EVO M-PCA	−0.13 ± 0.76	0.58	0.50	35%	50%	70%	70%
Haigis-TK	−0.28 ± 0.73	0.55	0.33	40%	65%	75%	80%
PEARL-DGS	0.00 ± 0.66	0.52	0.50	35%	50%	70%	90%

M-PCA = measured posterior corneal astigmatism. P-PCA = predicted posterior corneal astigmatism. TK = total keratometry.

## Data Availability

The raw data supporting the conclusions of this article will be made available by the authors on request.

## Abbreviations

The following abbreviations are used in this manuscript:

D	Diopters
RK	Radial keratotomy
PE	Prediction error
AL	Axial length
IOL	Intraocular lens
HOAs	Higher order aberrations
SD	Standard deviation
MAE	Mean absolute error
MedAE	Median absolute error
